# Grade III Solitary Fibrous Tumor/Hemangiopericytoma: A Rare Case of a World Health Organization Grade III Anaplastic Hemangiopericytoma

**DOI:** 10.7759/cureus.10926

**Published:** 2020-10-13

**Authors:** Matthew Jenson, Dalys Haymes, Jeet Patel

**Affiliations:** 1 Radiology, University of Florida College of Medicine, Jacksonville, USA

**Keywords:** solitary fibrous tumor, hemangiopericytoma, anaplastic hemangiopericytoma

## Abstract

Solitary fibrous tumors (SFTs) and hemangiopericytomas (HPCs) have been combined into a single designation in the most recent World Health Organization (WHO) guidelines as solitary fibrous tumor/hemangiopericytoma (SFT/HPC). These rare intracranial tumors can present as WHO grade I, II, or III tumors, with the risk of recurrence, metastasis, and mortality worsening with higher-grade tumors. We present a case of a patient with a WHO grade III SFT/HPC with an emphasis on the imaging features that help differentiate this type of tumor from meningiomas, which are much more common and can appear similar. Being able to help differentiate these tumors by their imaging appearance is important to help triage and risk-stratify patient management decisions.

## Introduction

In the most recent (2016) World Health Organization (WHO) Classification of the Central Nervous System, a new designation of solitary fibrous tumor/hemangiopericytoma (SFT/HPC) has been created in the mesenchymal, non-meningothelial tumor subgroup [1]. These tumors are of mesenchymal origin. As with many diseases, this reorganization was brought about by the discovery that these tumors have shared genetic origins with NAB2-STAT6 fusion [2-3]. Prior to this understanding, most literature had referred to SFTs and HPCs as distinct entities.

Tumors within the SFT/HPC designation can be WHO grade I, II, or III depending on pathologic features. WHO grade I SFT/HPC represent tumors with high collagen content and low cellularity, and correspond to tumors previously labeled as SFTs. WHO grade II SFT/HPC represents tumors with lower collagen content and higher cellularity with a vascular pattern typical of what was previously labeled as HPCs. WHO grade III SFT/HPC represents tumors that exhibit five or more mitoses per 10 high-power fields on microscopy, corresponding to what was previously labeled as anaplastic HPCs [4].

The age-adjusted incidence rate of SFT/HPC is 3.77 per 10,000,000 people [5]. Intracranial HPC (WHO grade II or III SFT/HPC) are estimated to account for 0.4% of all primary central nervous system tumors [6]. The presentation pattern of patients with SFT/HPC is nonspecific though headache is the most commonly described symptom [7].

## Case presentation

Our patient was a 65-year-old male who had presented to an outside facility with seizures. Upon further workup, he had been found to have a “brain tumor” on outside imaging and had been subsequently discharged on anti-epileptics and steroids. He had reported gradually worsening headaches and blurred vision and sought outpatient medical attention approximately one year following the initial seizure episode. Prior outside medical imaging was unavailable, and new imaging was acquired at our institution.

**Figure 1:**
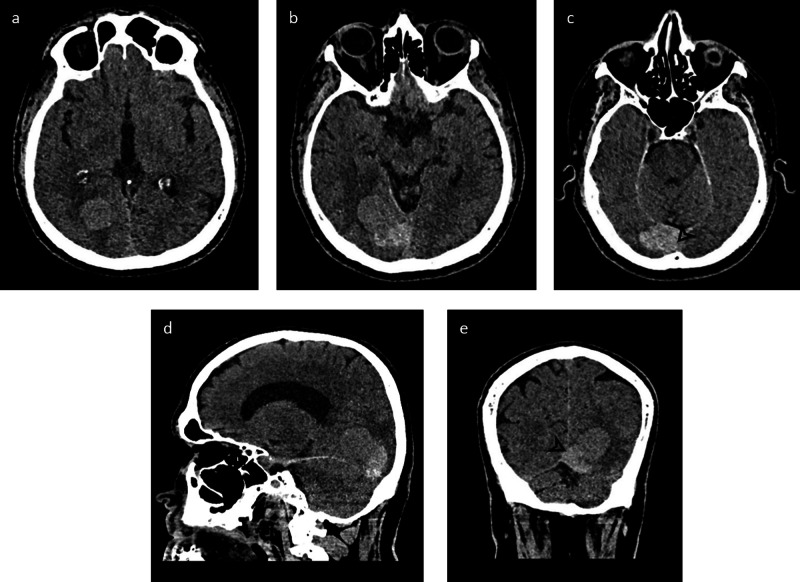
Non-contrast CT head Axial non-contrast CT head images along the superior (a), mid (b), and inferior (c) portions of the mass followed by sagittal (d) and coronal (e) reconstructions. There is a hyperdense mass, which is seen extending from the extra-axial space near the torcula along the posterior falx. There is mild surrounding vasogenic edema in the right occipital lobe. The mass exhibits a heterogeneous appearance with a relatively more dense component seen closer to its base with suspected dural venous sinus involvement (arrowhead) CT: computed tomography

**Figure 2:**
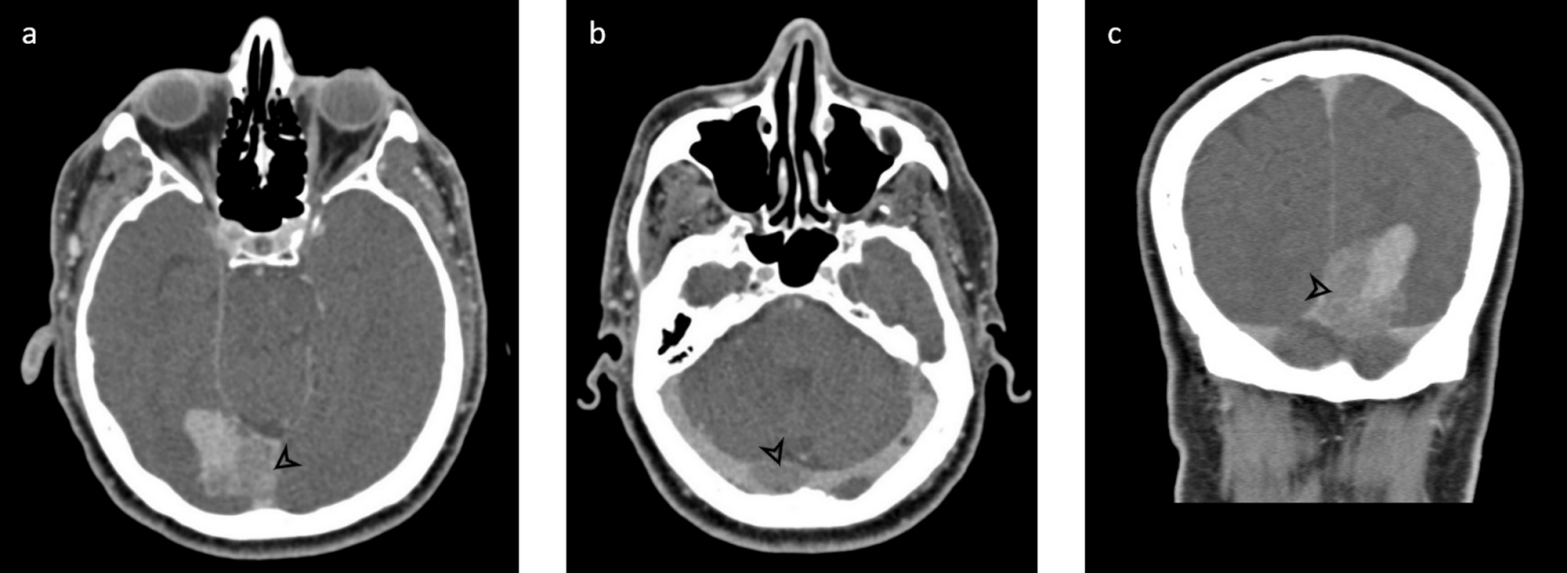
CT venogram Post-contrast CT venogram images along the middle (a) and inferior (b) portion of the mass, as well as coronal reconstruction (c). Tumoral invasion (arrowhead) is seen within the straight sinus, most posterior aspect of the superior sagittal sinus, torcula, and proximal right transverse sinus. The remaining portions of the transverse sinuses appear well opacified CT: computed tomography

The initial CT head without contrast demonstrated a dense right occipital, extra-axial mass with surrounding edema and concern for dural venous sinus involvement near the torcula (Figure 1). The mass appeared heterogeneous with areas of increased density near its base, along the region of the posterior falx. CT venogram confirmed tumoral involvement in portions of the straight sinus, torcula, superior sagittal sinus, and proximal right transverse sinus (Figure 2).

**Figure 3:**
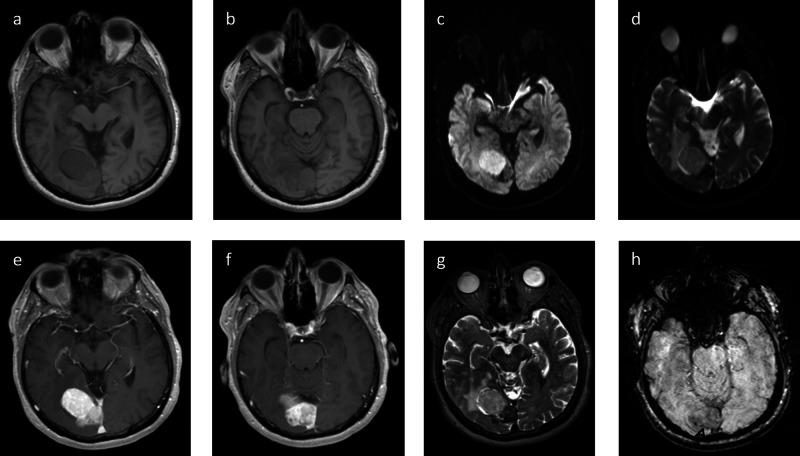
MRI Brain T1-weighted pre-contrast images along the superior (a) and inferior (b) aspects of the mass demonstrate mostly internal isointense signal though the main, somewhat pedunculated component exhibits a slightly T1-weighted hypointense signal relative to the base. Diffusion (c) and ADC (d) demonstrate restricted diffusion involving the main, rounded component. T1-weighted post-contrast images along the superior (e) and inferior (f) aspects of the mass demonstrate avid enhancement, though more homogenous enhancement is seen along the superior rounded component that restricts diffusion. The T2-weighted fat-saturated image demonstrates a hypointense signal along the base of the mass with a mildly hyperintense signal along the superior, rounded component. SWI sequence demonstrates areas of susceptibility artifact (arrowhead) along the base of the mass possibly related to blood products MRI: magnetic resonance imaging; ADC: apparent diffusion coefficient; SWI: susceptibility-weighted imaging

MRI demonstrated the T1-weighted isointense mass to avidly enhance and confirmed the dural venous sinus involvement. The region at the base of the mass that exhibited increased density on the non-contrast CT was found to exhibit a T2-weighted hypointense signal and more heterogeneous enhancement. Portions of the mass demonstrated restricted diffusion, and there was some susceptibility artifact seen along the base of the mass (Figure 3).

**Figure 4:**
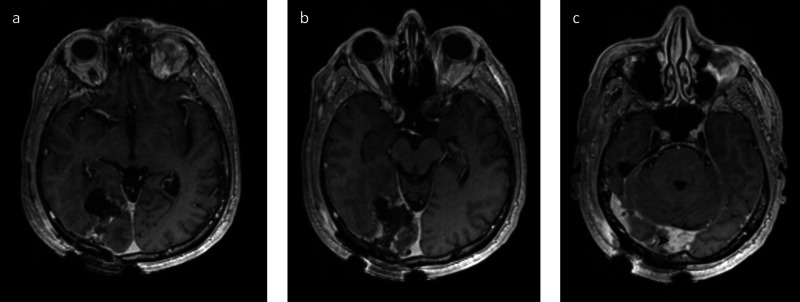
Postoperative MRI Postoperative T1-weighted post-contrast MP-RAGE images along the super (a), mid (b), and inferior (c) aspects of the resection cavity. The majority of the resection cavity exhibits thin peripheral post-contrast enhancement, likely post-surgical in nature. However, along the inferior aspect of the resection cavity, where the tumor was previously seen to involve the straight sinus, and torcula, there is a residual thickened enhancement (arrowhead) concerning for residual tumor, corresponding to an unresected tumor on the operative report MRI: magnetic resonance imaging; MP-RAGE: magnetization-prepared rapid gradient-echo

The patient underwent subtotal resection as portions of the mass that were invading the dural venous sinuses could not be fully resected, with a residual tumor in the dural venous sinuses demonstrated on postoperative MRI (Figure 4). The patient recovered well postoperatively and pathology results indicated WHO grade III SFT/HPC. Our institutional tumor board's recommendation was to proceed with adjuvant radiation and chemotherapy with imaging surveillance.

## Discussion

Central nervous system SFTs (WHO grade I SFT/HPC) are typically thought of as benign and treated with surgery alone [8]. Alternatively, HPCs (WHO grade II and III SFT/HPC) are more likely to recur and metastasize extracranially and therefore more often receive radiation therapy and/or chemotherapy in addition to surgical resection [9].

SFT/HPC tumors can often share imaging characteristics with meningiomas, though meningiomas are much more common and less likely to recur or metastasize extracranially [10]. HPCs (grade II and III SFT/HPC) are usually solitary, attached to the dura, are hypervascular with avid enhancement, and can cause adjacent edema, all of which are similar imaging characteristics to that of meningiomas. However, HPCs can result in bony erosion (as opposed to the hyperostosis frequently seen in meningiomas). Intratumoral calcification is usually not a feature of HPCs in contrast to meningiomas. Additionally, a mushroom appearance of the tumor with a relatively narrow base of attachment to the dura also favors HPCs over meningiomas, which typically have a broader dural base [7].

Now that the shared genetic basis of SFTs and HPCs has come to light, some studies have sought to retrospectively see if this new classification scheme would lead to different categorization of previously treated tumors and have re-analyzed survival data. For example, in 2018, Sung et al. retrospectively reevaluated 60 patients previously diagnosed with SFT/HPC-type tumors [11]. Upon this reevaluation, there were two cases previously diagnosed as SFTs that were regraded as grade II SFT/HPC, and one case previously diagnosed as SFT that was regraded as grade III SFT/HPC. Then, using the new WHO categorization scheme, they found that the five-year survival was 100% for grade II SFT/HPC vs. 71.8% for grade III SFT/HPC.

Due to our patient initially presenting at an outside facility, it was not clear what the planned follow-up had been after the initial discharge. However, given the patient’s worsening symptoms, further investigation and treatment were warranted. Understanding the different imaging features and risk of recurrence and metastasis between SFT/HPC tumors and meningiomas is important to help triage and stratify as to which cases should be more thoroughly investigated versus surveilled.

## Conclusions

SFT/HPC should remain an important differential consideration when working up extra-axial masses. Despite some similarities in imaging findings, there are often clues on imaging that can help lead to including SFT/HPC as a differential consideration. As these tumors exhibit different risk of recurrence and metastasis compared to much more common meningiomas, it is important to consider the possibility of an SFT/HPC in patients.
